# Recent Evidence of Hantavirus Circulation in the American Tropic

**DOI:** 10.3390/v6031274

**Published:** 2014-03-14

**Authors:** Carolina Montoya-Ruiz, Francisco J. Diaz, Juan D. Rodas

**Affiliations:** 1 Grupo Centauro, Universidad de Antioquia, Cll 70 No. 52-21, SIU 233, Medellín, Antioquia 050010, Colombia; E-Mail: carolinamontoyaruiz@gmail.com; 2 Grupo Inmunovirologia, Universidad de Antioquia, Cll 70 No. 52-21, SIU 532, Medellín, Antioquia 050010, Colombia; E-Mail: franciscodiaz314@gmail.com

**Keywords:** hantaviruses, new world, central America, South America, Caribbean, epidemiology, outbreak, hantavirus pulmonary syndrome, hemorrhagic fever with renal syndrome

## Abstract

Hantaan virus was discovered in Korea during the 1970s while other similar viruses were later reported in Asia and Europe. There was no information about hantavirus human infection in the Americas until 1993 when an outbreak was described in the United States. This event promoted new studies to find hantaviruses in the Americas. At first, many studies were conducted in Brazil, Argentina, Chile, Uruguay and Paraguay, while other Latin American countries began to report the presence of these agents towards the end of the 20th century. More than 30 hantaviruses have been reported in the Western Hemisphere with more frequent cases registered in the southern cone (Argentina, Chile, Uruguay, Paraguay, Bolivia and Brazil). However there was an important outbreak in 2000 in Panama and some rare events have been described in Peru, Venezuela and French Guiana. Since hantaviruses have only recently emerged as a potential threat in the tropical zones of the Americas, this review compiles recent hantavirus reports in Central America, the Caribbean islands and the northern region of South America. These studies have generated the discovery of new hantaviruses and could help to anticipate the presentation of possible future outbreaks in the region.

## 1. Introduction

Hantavirus is a genus in the family *Bunyaviridae*. These viruses infect humans through inhalation of excreta from persistently and asymptomatically infected rodents [1]. Some members of this genus are not pathogenic to humans, but others produce two distinct illnesses: (I) those found in Asia and Europe (old world hantavirus) cause hemorrhagic fever with renal syndrome (HFRS), which have a lethality approaching 12%, or epidemic nephropathy (EN) (a less severe form of the previous disease), with an approximate lethality of 1%; (II) the viruses circulating in the Americas (new world hantaviruses) generate a severe respiratory disease called hantavirus pulmonary syndrome (HPS), associated with a lethality between 10% and 50% depending of the virus genotype [2,3].

The viruses of this genus are oval or spherical particles with a diameter of approximately 100 nm [4]. The hantavirus genome consists of negative-sense, single stranded RNA. The long (L) segment encodes an RNA dependent RNA polymerase (RdRp); the medium (M) segment encodes the precursor of two glycoproteins, Gn and Gc; the short (S) segment encodes the nucleocapsid (N) protein and, in some cases, it also encodes a non-structural protein (NSs) in a superimposed open reading frame (ORF), which has been shown to be expressed in the Puumala (PUUV), Tula (TULV) and Andes (ANDV) viruses [5,6]. The 3' and 5' ends of each segment have complementary sequences that generate a closed circle. Within an infectious virion there is a copy of each of the three segments encapsidated by nucleocapsid protein and attached to the RdRp. The nucleocapsid is, in turn, wrapped in a lipid membrane where Gn and Gc are anchored and work as virion ligands that attach to the human integrins αIIaβ3, α5β1 and αvβ3 used as viral receptors [7].

The discovery of these viruses originated from a retrospective study of a HFRS outbreak during the Korean War (1950–1953). The etiologic agent was named Hantaan virus (HTNV), which was isolated from the wild rodent *Apodemus agrarius.* HTNV is considered the virus prototype of the new genus Hantavirus [8,9]. Subsequent to this finding, other viruses of the same genus were identified in Asia and Europe, which also caused HFRS or NE.

The first hantavirus identified in the new world was Prospect Hill virus (PHV) isolated from *Microtus pennsylvanicus* in Maryland (United States) but no disease in humans has been associated with this agent [8,10]. Subsequently, in May, 1993 an outbreak of a high-mortality respiratory disease occurred in the Four Corners region (southwest United States); the clinical presentation was characterized by fever, myalgia, headache and cough, followed by respiratory failure. Subsequent investigations identified the etiologic agent as a new virus, which was called Sin Nombre virus (SNV). The deer mouse *Peromyscus maniculatus* was identified as the reservoir of SNV and the disease was named HPS [3,11].

**Figure 1:**
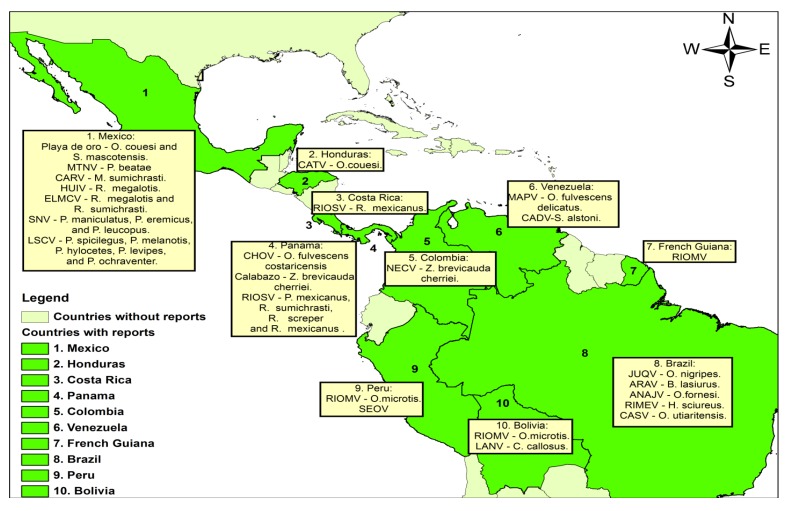
Reported hantaviruses in tropical American countries. Map by Leidy Y Acevedo**-**Gutiérrez with ArcGIS v10.0. [35]. ANAJV, Anajatuba virus; ARAV, Araraquara virus; CADV, Caño Delgadito virus; CARV, Carrizal virus; CASV, Castelo dos Sonhos virus; CATV, Catacamas virus; CHOV, Choclo virus; ELMCV, El Moro Canyon virus; HUIZ, Huitzilac virus; JUQV, Juquitiba virus; LANV, Laguna Negra virus; LSCV, Limestone Canyon virus; MAPV, Maporal virus; MTNV, Montano virus; NECV, Necocli virus; RIMEV, Rio Mearim virus; RIOMV, Rio Mamore virus; RIOSV, Rio Segundo virus; SEOV, Seoul virus; SNV, Sin Nombre virus.

The discovery of SNV promoted the search for the presence of these agents in other parts of the continent, and surveillance for possible new cases. This resulted in the discovery of many new hantaviruses that, at present, total more than 30 genotypes reported in the Americas. Most of these genotypes had been recovered from rodent hosts and have not been associated with human disease. However, sporadic cases of HPS have been diagnosed in Argentina, Peru, Brazil, Chile, Uruguay, Panama, Bolivia, Venezuela, Paraguay and French Guiana [11,12,13] (Table 1 and Figure 1). 

**Table 1:** Evidence of hantavirus circulation in rodents from tropical American countries.

Country	Genotype	Acronym	Associated Host	Reference
Mexico	Playa de Oro		*Oryzomys Couesi* and *Sigmodon mascotensis*	[14]
	Montano	MTNV	*Peromyscus beatae*	[15]
	Carrizal	CARV	*Megadontomys sumichrasti*	[15]
	Huitzilac	HUIV	*Reithrodontomys megalotis*	[15]
	Moro Canyon	ELMCV	*Reithrodontomys megalotis* and *R. sumichrasti*	[16]
	Sin Nombre	SNV	*Peromyscus maniculatus*, *P. eremicus,* and *P. leucopus*	[16]
	Limestone Canyon	LSCV	*Peromyscus spicilegus*, *P. melanotis*, *P. hylocetes*,* P. levipes and P ochraventer*	[16]
Honduras	Catacamas	CATV	*Oryzomys* *couesi*	[17]
Costa Rica	Rio segundo	RIOSV	*Reithrodontomys mexicanus*	[18]
Panamá	^a ^Choclo	CHOV	*Oligoryzomys fulvescens costaricensis*	[19,20]
	Calabazo		*Zygodontomys brevicauda cherriei*	[20,21]
	Rio Segundo	RIOSV	*Peromyscus mexicanus*,* Reithrodontomys sumichrasti*,* R. screper* and *R. mexicanus*	[21]
Colombia	Necoclí	NECV	*Zygodontomys brevicauda cherriei*	[22]
Venezuela	Maporal	MAPV	*Oligoryzomys fulvescens delicatus*	[19]
	Caño Delgadito	CADV	*Sigmodon alstoni*	[23]
French Guiana	^a ^Rio Mamoré	RIOM	Human case	[24]
Bolivia	^b ^Rio Mamoré	RIOMV	*Oligoryzomys microtis*	[25,26]
	^a ^Laguna Negra	LANV	*Calomys callosus*	[26]
	^c ^Bermejo	BMJV	Human case	[27]
Perú	^a ^Rio Mamoré	RIOMV	*Oligoryzomys microtis*	[28,29]
	^a ^Seoul	SEOV	Human case	[30]
Brazil	^a ^Juquitiba	JUQV	*Oryzomys nigripes*	[31,32]
	^a ^Araraquara	ARAV	*Bolomys lasiurus*	[31,32]
	^a ^Castelo dos Sonhos	CASV	*Oligoryzomys utiaritensis*	[31,33]
	Anajatuba	ANAJV	*Oligoryzomys fornesi*	[34]
	Rio Mearim	RIMEV	*Holochilus sciureus*	[34]

^a^ Associated with human disease reported in that country; ^b^ Viral sequence from patient was not available but the suspected etiological agent is RIOMV; ^c^ It was not possible to confirm the geographic location where patient got infected.

It has been proposed that hantavirus species must differ by more than 7% in their amino acid sequence of S and M segments. Although genotypes described so far differ widely in their degree of sequence similarity, in order to consider an isolate a new species by ICTV, a rigorous genetic, ecologic and antigenic characterization is required. Thus, some of the hantaviruses mentioned here are not real species but strains; in some reports a new strain of a hantavirus species was assigned a different name, such as Maripa virus, which is possibly a strain of Rio Mamore virus [13].

**Figure 2:**
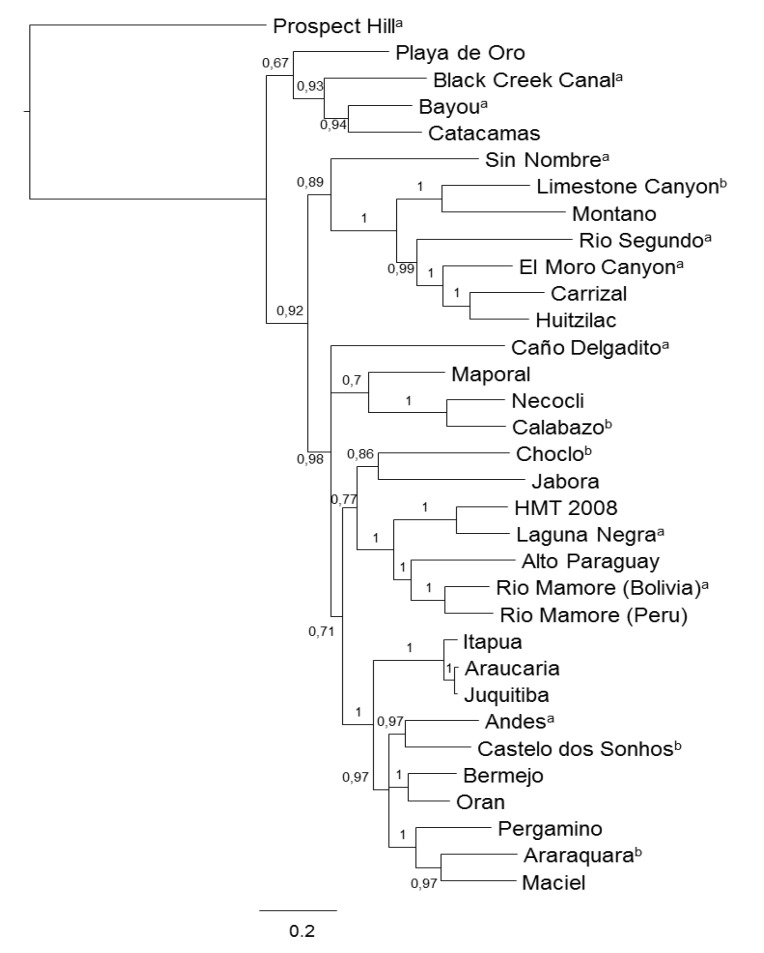
Bayesian phylogenetic analysis of the S segment genome of the Hantaviruses found in the Americas. Two parallel one million generations analyses were run sampling one in every 1000 states. The GTR plus gamma substitution model was used. The resulting majority-rule consensus tree was rooted with the sequence of Prospect Hill virus. Numbers close to nodes are posterior probabilities of the corresponding clades. The analysis was performed with MrBayes v3.2.2 program [37] and the tree was drawn with the FigTree v1.1.2 [38] package. a–b, Recognized and tentative species into genus Hantavirus by the 9th report of the ICTV respectively.

A phylogenetic analysis of the S segment sequences shows that hantaviruses from South America and Panama are monophyletic with respect to those from North America (Figure 2). This suggests that hantaviruses migrate from North to South America, perhaps with the arrival of *Sigmodontinae* rodents when the Isthmus of Panama was formed.

It is worth mentioning that countries of the southern cone of South America (Argentina, Brazil and Chile) are at the forefront of hantavirus research in Latin America [36]. Accordingly, surveillance programs followed by health and science institutions in these countries have had a greater capacity to identify possible cases, and have accumulated more knowledge about the circulation of hantaviruses in their territories. Conversely, the northern countries in South America such as Colombia, Bolivia, Ecuador, Venezuela, Peru and the countries of Central America and the Caribbean, have only started conducting research in the past twenty years, and thus knowledge about hantavirus circulation is still emerging. With this in mind, this review aims to compile the evidence of hantavirus circulation and the cases presented in these countries starting in the year 2000.

## 2. Reports of Hantaviruses in the Northern Cone of South America and the Brazilian Amazon

### 2.1. Venezuela

Since 1997, hantavirus circulation in Venezuela was evident with the isolation of Maporal virus (MAPV) from the *Oceomys bicolor* rodent, captured in the center of Venezuela. Although not associated with human illness, it was observed that inoculation of a Syrian golden hamster (*Mesocricetus auratus*), with MAPV reproduced the symptoms of HPS, making this virus a model for the study of HPS pathogenesis [39]. 

In 1997, a rodent sampling was performed on the Venezuelan grasslands in order to detect any hantaviruses. From this study, the analysis of viral sequences obtained from the cotton rat *Sigmodon alstoni* rodent resulted in the discovery of another hantavirus named Caño Delgadito virus (CADV) [23].

In 1999, the first human case of hantavirus infection in Venezuela emerged, confirmed by serological tests for IgG and IgM, though information about the etiology of this particular virus is not available. Nevertheless, a human seroprevalence study was conducted after this report. This study analyzed 1,380 samples, from which 173 were collected in Caño Delgadito (Portuguesa), 367 in Caracas, 29 in Carabobo, 76 in Lara, 96 in Tachira and 64 from military recruits. Another 328 sera were collected from pregnant women from Anzoategui, and 246 more from Amerindian living in Zulia. The results displayed a prevalence of 1.7%, and the individuals from Carabobo showed a significantly higher seroprevalence than the entire population (10.3%) [40].

There has also been some research on the characterization of the interactions of these two viruses with their hosts. One particular study evaluated whether *Sigmodon alstoni* behaved as a reservoir for CADV. In this case, rodents were inoculated with the viral isolate detecting viral RNA at 54 days post infection. It was also possible to recover infectious virions from different secretions, suggesting the development of a persistent infection and concluding that *S. alstoni* acts as a reservoir for this virus [41].

Years later, a study obtained viral sequences from 280 rodents captured on farms in western Venezuela, all the sequences corresponding to the CADV originating from the *Sigmodon alstoni* rodent and all sequences from *Oligoryzomys sp* grouped with the MAPV. There were no sequences of these two viruses present in any other species, suggesting that these two rodents are the main hosts for both viruses [42]. In this report the authors discussed the difficulty of distinguishing between species of *Oligoryzomys* genus and accordingly, in a subsequent study, they analyzed the Cytochrome b sequences of Choclo virus (CHOV)-infected *Oligoryzomys* rodents from Panama, and MAPV-infected *Oligoryzomys* from Venezuela. This work found that *Oligoryzomys costaricensis* is associated with the CHOV and *Oligoryzomys delicatus* is associated with the MAPV [19].

### 2.2. Colombia

In 2004, the first report supporting evidence of hantavirus circulation in Colombia was published. This work evaluated the presence of reactive IgG against the SNV antigen in a population of rural workers from the states of Cordoba and Sucre, and detected a seropositivity prevalence of 13.5% [43]. The same authors evaluated 336 rodent samples from 11 municipalities of the Cordoba department, finding a seroprevalence of 2.1%, where the majority of seropositive rodents belonged to the *Sigmodontinae* subfamily [44]. 

Later, Londoño *et al*., carried out a study in northwestern Antioquia department where they captured 354 rodents and evaluated for the presence of IgG reactive for SNV and Maciel virus (MCLV), finding 15 positive rodents belonging to the species *Zygodontomys* *brevicauda* cherriei [22]. Tissues from seropositive rodents were used to obtain viral sequences by RT-PCR. Eleven samples tested positive for hantavirus S and M segments and phylogenetic analysis of those sequences revealed that they belonged to a virus closely related to Calabazo, a hantavirus previously reported in the Azuero peninsula of Panama. They called this, apparently new, virus Necocli after the municipality where rodents were captured. Necocli is not geographically distant from Panama; however, the viral characterization has not yet been performed (Figure 1 and Figure 2) [22].

### 2.3. Ecuador

Despite previous reports in neighboring countries, no studies in Ecuador indicate circulation of these viruses. The only publication speculating its presence is the case of a Belgium tourist who developed epidemic nephropathy from hantavirus after his return from a trip to Ecuador. In that patient, it was initially thought that he was infected by the Seoul virus (SEOV), the only old world hantavirus whose circulation has been established in the new world [45]. Later on, a fragment of the viral genome was amplified from this patient and the corresponding sequence was identified as PUUV, an old world hantavirus, whose reservoir is *Myodes glareolus.* Since this rodent has not been detected in the Americas, the authors concluded that this patient was possibly infected on his return to Belgium [46].

### 2.4. Bolivia

In Bolivia, hantavirus circulation was evident in 1996 when the Rio Mamore virus (RIOMV) was described through characterization of sequences obtained from the rodent *Oligoryzomys microtis* [25]. In 1998, the first HPS case was reported in a Chilean patient who acquired the infection during a trip to Bolivia. Partial sequences obtained from the samples of this patient showed a nucleotide sequence identity of 84% and 87% with Gn and nucleocapsid sequences of Laguna Negra virus (LANV), previously reported in Paraguay, suggesting that the hantavirus responsible for this case had possibly been a Laguna Negra variant [47,48].

Between May and July of 2000, two human cases of HPS in Bermejo City were confirmed by IgG and IgM ELISA assay and RT-PCR. Viral sequences obtained from the first patient indicated that the etiological agent belonged to a previously reported Andes North linage [49], and those obtained from the second patient were classified as Bermejo virus (BMJV) previously detected in *Oligoryzomys chaconesis* in Oran city (Salta, Argentina). Although the second patient lived in Bolivia, he was working as a muleteer in Salta province (Argentina) and returned to Bolivia three weeks before symptoms appeared; therefore it is unclear where he acquired the infection [27]. 

In August 2002, two human cases of HPS were confirmed in Mineiros and Concepcion in the state of Santa Cruz. A study carried out by Carroll, *et al*., in 2005 described the presence of hantavirus antibodies in the rodent *Oligoryzomys microtis* captured in Mineiros and *Calomys callosus* captured in Concepcion [26]. The authors amplified viral sequences and observed that the sequences obtained from *O. microtis* showed 90% identity with RIOMV and those from *C. callosus* showed 87% to 88% identity with LANV. Those obtained from the sample of the second patient showed 99% identity with those obtained from *C. callosus*, indicating that this rodent is the host for LANV, responsible for the HPS case near Concepcion. The samples of patients from Mineiros were not available for comparison with the rodent sequence. This result is consistent with the hypothesis that *C. callosus* y *C. laucha* (the reservoir of LANV in Paraguay) show a high genetic similarity, potentially indicating that this virus evolved from a common ancestor [26]. 

With the goal of performing a better description of the genetic diversity of Bolivian hantaviruses, Cruz *et al*., sampled febrile patients suspected of having suffered recent hantavirus infection in Chapare Province in 2008–2009 [50]. Viral RNA was detected in three patients and the partial sequences of the S and M segments showed high correlation with the Andes virus linage. In the same study they also evaluated IgG against hantavirus using antigens from ANDV and LANV and found a seroprevalence of 12.2% [50]. In summary, it seems that up to four hantaviruses could be present in Bolivia: RIOMV, LANV, BMJV, and ANDV.

### 2.5. Peru

In 1996, a variant of RIOMV was found in *Oligoryzomys microtis* rodents captured in Iquitos, capital of the Maynas Province (Loreto, Peru). This virus was originally called HTN-007 [28], though it was not clearly stated whether it was a new hantavirus or a strain of RIOMV, which had been initially detected in a very distant location [25]. Sequencing of a large part of the genome, including a fragment of the L segment, demonstrated a high degree of identity, indicating that it was a strain of RIOMV, thus the geographic range of this virus was extended from Bolivia to northern Peru [51].

In 2011, two fatal cases with clinical suspicion of HPS and HFRS occurred in Loreto, in the Amazon region of Peru. The first case was a 29-year-old woman, who visited locations along the Nanay River working as a tourist guide. This person showed fever, headache, and acute pulmonary edema requiring hemodialysis days before her death. The second case was a 33-year-old woman who died in a hospital in Iquitos; she developed fever, headache, myalgia, hemorrhagic manifestations, acute renal failure with liver involvement, and cardiovascular and lung damage. In both cases the diagnoses were confirmed by the detection of IgM and RT-PCR amplification of 540nt of the M segment, which showed 97% homology with the SEOV [30]. This agent is the only old world hantavirus whose circulation had been previously reported in the Americas [45,52].

Two additional cases of HPS were reported that year near the city of Iquitos; both were clinically characterized by fever, malaise, abdominal pain, watery diarrhea, coughing, cyanosis and severe acute respiratory failure, which evolved to renal failure and refractory shock. In both patients the causative agent was confirmed by molecular tests, which demonstrated 96% similarity with the RIOM variant previously detected in *Oligoryzomys microtis* in the same location [29].

A retrospective study was performed on febrile patients tended to in 12 health centers from 2007 to 2010 in the Maynas province in order to find serological evidence of recent hantavirus infection. Fifteen out of 5,174 patients, who had suffered a relatively mild disease, were found to be IgM reactive to ANDV antigen. In the same study, a cross-sectional analysis of healthy residents of Iquitos revealed a seroprevalence of 1.7% (33/2063) [53].

### 2.6. French Guiana

The presence of hantavirus circulation in French Guiana was suggested in a retrospective serologic survey which evaluated the seroprevalence in 420 patients with compatible symptoms with HPS, finding that seroprevalence of IgG was 1.42% and none of the samples were positive for IgM [54]. A HPS case was reported in 2008 with seropositive diagnosis by IgM and IgG detection and confirmed by viral genome amplification. Analysis of the sequence obtained suggested that the etiologic agent was RIOMV or a closely related virus, which was named the Maripa virus [13]. Recently viral sequences were obtained from this patient and compared with RIOMV showing very high amino acid identities 97.7%, 96.4%, and 95.8% for N, Gn/Gc and L proteins respectively suggesting that the Maripa virus is a new strain of RIOMV [24].

### 2.7. Brazil

According to the scientific literature, increasing numbers of studies performed in recent years in Brazil report on the circulation of hantavirus. In this review we will focus only on those studies conducted in the northern and Amazonian region of Brazil.

The first case of HPS in Brazil was reported in 1993 in the state of São Paulo; the causative agent was identified as Juquitiba virus (JUQV) [55]. Other cases were detected soon after in different parts of the country. Samples from some of those patients were used to perform RT-PCR and sequencing, which lead to the discovery of two new hantaviruses, named Araraquara virus (ARAV) and Castelo dos Sonhos virus (CASV) after the towns where the cases occurred [31]. 

By 2004, the number of diagnosed cases of HPS in Brazil had increased to 342, most being located in the south and southeast of the country. Some cases were also found in the sates of Minas Gerais (54), Mato Grosso (33), Maranhao (7), Para (4), Goias (3), Rio Grande do Norte (1) and in Bahia (1) [32]. Considering this large number of cases, Suzuki *et al*., conducted a genetic analysis of viral sequences from patients and rodents captured in the presumed sites of infection [32]. Partial sequencing of M and S segments obtained from 11 HPS patients and seven rodents indicated that ARAV and JUQV virus were circulating in Atlantic and Cerrado areas located in the southern states of Parana, Santa Catarina, and Rio Grande do Sul and at the southeastern states of Minas Gerais and São Paulo. They also demonstrated that *Bolomys lasiurus* and *Oryzomys nigripes* were the hosts of ARAV and JUQV, respectively [32].

In the Amazon region of Brazil, circulation of hantavirus was reported in 2004; the first cases of HPS were reported in four patients, all inhabitants of a rural area near Itacoatiara. All of these patients belonged to same family that had cleared a forest near their farm and were declared to have been in contact with many rodents. All of the cases but one were confirmed by the IgM ELISA test [56].

A cross-sectional study in Maranhão between 2004 and 2006 evaluated potential risk factors for hantavirus infection in this region. Samples were obtained from 1,389 inhabitants from this area and tested for IgG presence against SNV antigen. The seroprevalence detected was 4.7%, with most of the positive samples from farmers [57]. In the same locations, rodent sampling was performed which resulted in the identification of two potentially new hantaviruses, Anajatuba (ANAJ) and Rio Mearim sequences (RIME), which were detected in *Oligoryzomys fornesi* and *Holochilus sciureus*, respectively [34].

Another study published in 2010 estimated the seroprevalence of antibodies reactive to a recombinant capsid antigen of ANDV in different areas in the Amazon area and found 2.16% in Novo Progresso, 4.37% in Trairão (state of Para), 4.74% in Guarantã do Norte and 9.43% in Marcelândia (state of Mato Grosso). The Other studies confirmed the circulation of CASV virus in samples of patients with HPS through viral genome amplification and detect its likely principal rodent host *Oligoryzomys utiaritensis* [33,58].

The human seroprevalence for hantavirus in the Amazon state was estimated with samples collected from 2007 to 2009. This was determined by ELISA assay using recombinant nucleocapsid protein from ARAV. About 0.6% (10/1731) of the samples were positive for IgG and none of the same samples were positive for IgM. The positive individuals belonged to the municipalities of Atalaia do Norte, Careiro Castanho, Itacoatiara and Labrea indicating exposure to hantavirus throughout the entire state [59].

## 3. Reports of Hantavirus Circulation in Mexico, Central America and the Caribbean Islands

### 3.1. Mexico

Unlike the New Mexico panorama where the first HPS cases reported resulted in the discovery of SNV, Mexico has not registered any case of HPS despite various studies conducted in its territory [14]. This situation could be explained by deficiency in diagnostic resources and the lack of preparedness of the medical staff, which could confuse these cases with other diseases with similar clinical pictures. However different hantaviruses have been described in this country, such as the Playa de Oro virus carried by *Oryzomys Couesi* and *Sigmodon mascotensis* identified in Colima, southwestern Mexico, and the El Moro Canyon virus (ELMCV) carried by *Reithrodontomys megalotis*, initially detected in the United States [14,60]. Additionally, serological evidence has been detected in some rodents such as *Reithrodontomys mexicanus*, *Reithrodontomys sumichrasti*, *Peromyscus maniculatus*, and *Peromyscus levipes* [61,62].

Recently genetic evidence of hantavirus circulation in Mexican rodents was published. The first study captured 211 rodents in Guerrero and Morelos states and reported three new hantaviruses: Montano (MTNV), Carrizal (CARV) and Huitzilac viruses (HUIV) in *Peromyscus beatae, Megadontomys sumichrasti*, and *Reithrodontomys megalotis*, respectively [15]. The second study evaluated the seroprevalence in 876 rodents (44 species from *Neotominae* subfamily and 10 species from *Sigmodontinae*), sampled from eight different states. The seroprevalence in this study was 4.0% and samples from seropositive rodents were used to amplify viral genomes. Five rodents were positive for SNV sequences indicating that this virus is enzootic in Nuevo León, San Luis Potosí, Tamaulipas and Veracruz states. Viral sequences obtained from *Reithrodontomys megalotis* and *R. sumichrasti* indicated ELMCV circulation, and sequences obtained from *Peromyscus spicilegus*, *P. melanotis*, *P. hylocetes*, *P. levipes* and *P. ochraventer* indicated the presence of Limestone Canyon (LSCV) virus and other hantaviruses were widely distributed throughout Mexico [16].

### 3.2. Panama

In 1999, suspected HPS cases were reported in the Azuero peninsula, from patients whose diagnostics were confirmed by serologic testing and viral genome detection. Sequences obtained from S and M segments were used in phylogenetic analysis and indicated that these patients were infected with a new hantavirus denominated as Choclo virus (CHOV). This was the first evidence of pathogenic hantavirus circulation in this country [20]. Subsequently, sequences of this hantavirus were amplified from samples of *Oligoryzomys fulvescens* rodents captured in the same place. The viral sequence obtained from these rodents had a high similarity to the one obtained from patients with HPS, indicating that this rodent is a host for CHOV. Additional sequences were obtained from *Zygodontomys brevicauda*, but these belonged to another hantavirus, which was later named the Calabazo virus [20]. One important observation regarding HPS cases produced by CHOV was that they all showed a pulmonary disease similar to the one produced by SNV, however the former has a lower lethality rate (10%) compared with the one associated with other new world hantaviruses [63].

A further study was performed to identify populations of small mammals with potential importance in the epidemiology of HPS in Panama. Researchers sampled 556 rodents and marsupials from central and western Panama from 2000 to 2002 and evaluated the presence of antibodies (IgG) against hantavirus. The results indicated that 2.7% of animals were positive. This study also reported sequences of another hantavirus previously unidentified in Panama, that was called Rio Segundo virus (RIOSV). Seroprevalences for hantavirus in *Oligoryzomys fulvescens* and *Zygodontomys brevicauda* in the Azuero peninsula were 0.06% (4/72) and 0.05% (5/108) respectively suggesting that the former could be the principal host for CHOV and the Calabazo virus [21]. Conversely, in a different report, Cytochrome b gene sequence analyses was used to reclassify the rodent species infected with CHOV and MAPV, and indicated that the main host associated with CHOV is *Oligoryzomys costaricensis* and not *Oligoryzomys fulvescens* as previously mentioned [19].

An ecological study also demonstrated that the occurrence of the Panamanian outbreaks was favored by agricultural activity, especially for rice production, and the biodiversity reduction of wild rodents, supporting the increase of the population density in the rodents known as hantavirus main hosts [64,65]. From 2001–2007 Armien *et al*., performed several hantavirus seroprevalence studies in different communities: three in the Santos province and one in the Veraguas providence, and the prevalence of these agents varied between 16.5% and 60.4% [66].

### 3.3. Other Central American Countries

Except for Panama, most of Central American countries have not performed sufficient studies regarding hantavirus circulation. Some countries have limited information and others have not yet begun this work. 

Research in Central America began in 1995, and soon after the discovery of SNV, a new Hantavirus called RIOSV was identified in rodent samples of *Reithrodontomys mexicanus* from Costa Rica [18]. Another study made in 2006 in Honduras evaluated the seroprevalence in rodents captured in Catacamas, and found 20.8% (5/24) *Oryzomys couesi* seropositive for hantavirus. In this study, viral isolation from lung tissue of one seropositive rodent was successful and the agent was characterized as a new virus closely related to Bayou virus (BAYV) and named the Catacamas virus (CATV) [17].

## 4. Caribbean Islands

Evidences of hantavirus circulation has been mostly limited to the continent. The first report from a Caribbean island was published in Barbados in 2002. This research found hantavirus IgM and IgG serological evidence in patients with suspected leptospirosis. Later serological evidence was also detected in *Rattus norvegicus*, an identified SEOV host, indicating that SEOV or other related hantaviruses might be present in Barbados [67]. In Trinidad and Tobago, a study evaluated the seroprevalence in healthy workers of livestock farms and slaughterhouse workers finding an 11.4% seroprevalence [68].

## 5. Conclusions

During recent years, the understanding and diagnoses of hantavirus infections have improved notably in the Americas. Unlike the southern cone of South America and Brazil that have developed many studies about hantavirus circulation immediately after the discovery of SNV [3], other tropical countries of South and Central America began their research after the year 2000, or they have not even started their own characterization regarding hantavirus circulation. Many of these studies resulted in the identification of new possible hantaviruses [17,20,22,25,33,49,51,69,70], which have unearthed evidence that hantaviruses are distributed worldwide. If countries have not documented its presence, it is very likely due to their lack of research. 

Supporting this affirmation, many studies have reported serological evidence of Hantavirus circulation in rodents and humans, the first one showed a broad diversity in likely primary hosts in these countries. This emphasizes the importance of improving the knowledge about the ecology of these species and characterizing human activities that generate risk to get hantavirus infection (Table 2). In addition, the serological evidence in human populations suggest a constant exposure in different countries. In general the studies show low frequencies (approximately 0.6% to 13.5%) and only a recent study shows high values 60.4% [66]. These differences could be associated with the particular features of the sampled population such as age, occupation (rural or urban worker) or to be a febrile patient. However, these results should be analyzed according to the antigen used in the employed serologic test that could reduce or improve the sensibility finding cross reactivity to closely related phylogenetic antigens (Table 3).

Although there are many studies that provide some evidence for new hantaviruses, most reports have not carried out the assays that fulfill the requirements of the International Committee for the Taxonomy of Viruses (ICTV) to define a new species. These conditions are: (I) a divergence of the amino acid sequence higher than 7% in comparison with the previously reported viruses that is supported by the complete sequences of their segments S and M; (II) at least four-fold difference on a cross sero-neutralization assay; (III) the new virus candidate must not be a product of genetic reassortment between different previously identified hantavirus strains; and (IV) the new virus should occupy a unique ecological niche with a specific primary reservoir [71]. Studies fulfilling these conditions would support the knowledge about the circulation of these viruses in different territories, the description of the genetic and antigenic diversity, their evolution and the evolution of their hosts. These would in turn contribute to knowledge of their ecology and epidemiology, allowing for predictions of the risk of appearance of new outbreaks in different localities.

This landscape would allow us to determine whether the frequency of HPS infection is truly increasing in Latin America. It is noteworthy that the lack of clinical cases in some countries is directly proportional to the absence of research performed on this area. This is probably the case in most of the tropical countries of Central and South America, since HPS and HFRS are not considered a differential diagnosis in compatible human cases, and also because of the lack of diagnostic tools needed to confirm the suspected cases. As a consequence, new studies are needed that improve the knowledge about the dynamic of circulation of these new viruses that allow us to reveal possible new hantaviruses and to increase the awareness among medical care centers, physicians and other health care professionals. We also need studies that help us to build infrastructure for research on this topic in developing countries, which also happen to be the places where most emerging viruses are thriving [72], and identify risk factors in exposed populations and geographical distribution of new hosts and reservoirs.

**Table 2:** Frequency of seropositive rodents in tropical American Countries.

Country (State/Providence)	Positive/Tested by Rodent Species ^a^, (Overall Percentage), Antigent ^b^	Ref.
Mexico (Colima )	23/358 *Oryzomys Couesi*, 6/87 *Sigmodon mascotensis* *and* 1/77 *Baiomys musculus*, (5%)	[14]
Mexico (DF and Jalisco)	1/8 *Peromyscus maniculatus* and 1/1 *Reitrodontomys sumichrasti* (6%)	[61]
Mexico (Tamaulipas)	7/31 *Peromyscus levipes*, (7/31)	[62]
Mexico (Morelo and Guerrero)	17/50 *Peromyscus beatae*, 1/6 *Megadontomys thomasi*, 1/6 *Neotoma picta*, 6/15 *Reithrodontomys sumichrasti*, and 2/25 *R.megalotis*, (12.7%)	[15]
Mexico (18 States)	1/43 *Baiomys musculus*, 1/48 *B. taylori*, 1/10 *Oryzomys Couesi*, 1/19 *Peromyscus eremicus*, 2/8 P. hylocetes, 2/15 *P. leucopus*, 4/51 *P. levipes*, 3/20 *P. maniculatus*, 1/29 *P. megalops*, 3/135 *P. melanotis*, 2/11 P. ochraventer, 2/31 P. spicilegus, 1/43 Peromyscus spp., 5/45 *Reithrodontomys megalotis*, 1/4 R. microdon, 5/23 R. sumichrasti, (4%), CADV	[16]
Costa Rica (Heredia, Cartago and Puntarenas)	1/3 *Reithrodontomys mexicanus*, (33%) ^c^	[18]
Honduras (Olancho)	5/24 *Oryzomys couesi*, (20.8%) CADV	[17]
Barbados (Bridgetown)	19/68 *Rattus Norvegicus*, (25.3%), mix of old world hantaviruses	[67]
Panama (Los Santos)	4/50 *Zydodontomys brevicauda* and 2/15 *Oligoryzomys fulvenses* (then it was reclassify in *O.fulvescens costaricensis*), (5%)	[20]
Panama (Los Santos)	5/108 *Zydodontomys brevicauda*, 4/72 *Oligoryzomys fulvescens costaricensis*, 1/22 *Peromyscus mexicanus*, 3/4 *Reithrodontomys sumichrastis*; 1/7 *R. Mexicanus*, 1/2 *R. creper*, (27%)	[21]
Venezuela (Cojedes, Portuguesa, Barinas)	1/13 *Oryzomys bicolor*, 10/166 *Sigmodon alstoni*, 1/45 *Zygodontomys brevicauda* and 1/29 *Rattus rattus*, (5%), PHV	[20]
Colombia (Cordoba)	1/17 *Heteromys* sp., 4/47 *Oryzomys* sp., 1/11 *Oligoryzomys* sp. and 1/2 (50%) *Proechimys* sp., (2,1%)	[44]
Colombia (Antioquia)	15/109 *Zygodontomys brevicauda cherriei*, (4.2%), SNV and MCLV	[22]
Peru (Loreto)	12/50 *Oligoryzomys microtis*, (24%)	[28]
Brazil (Maranhão)	1/40 *Bolomys lasiurus*, 5/12 *Oligoryzomys fornesi* and 15/52 *Holochilus sciureus*, (20.2%), SNV and ANDV	[34]

^a^ Non-positive species were excluded from this table; ^b^ All the studies used SNV antigen except otherwise specified; **^c^** This is part of a bigger study.

**Table 3:** Frequency of seropositive humans in tropical American Countries.

Country (State/Province)	Frequency of Seropositive/Tested (%), Ig ^a^, antigen ^b^	Studied Population	Ref.
Barbados (Bridgetown)	11/60 (18%) IgM and 4/60 (6,6%) IgG, using mix of old world hantaviruses	Leptospiroses-like cases	[67]
Trinidad and Tobago	27/236 (11,4) IgG, mix of old and new world hantaviruses	Healthy abattoir and livestock farm workers	[68]
Panama (Los Santos and Veraguas)	371/1129 (32.9%) IgG	Healthy general population	[66]
Venezuela (Portuguesa, Capital region, Carabobo, Lara, Tachira, Anzoategui, Zulia, some samples from military recruits)	80/1380 (5.8%), IgG, New York virus	Healthy general population	[40]
Colombia (Cordoba)	12/88 (13.5%), IgG	Healthy rural volunteers	[43]
Bolivia (Chapare)	61/500 (12.2%) IgG	Healthy general population	[50]
Bolivia (Chapare)	9/372 (2.4%), IgG, ANDV or LANV	Febrile patients	[50]
Peru (Loreto)	15/5175 (0.2%) IgM and all samples were negative for IgG, ANDV	Febrile patients	[53]
Peru (Loreto)	36/2063 (1.7%), IgG, LANV, SNV, RIOMV	Healthy general population	[53]
French Guiana	All samples were negative for IgM and 6/420 (1.4%) had IgG	Febrile patients	[54]
Brazil (Maranhão)	65/1389 (4.7%) IgG	Healthy general population	[57]
Brazil (Pará)	3/2737 (0.1%) IgM and 148/2737 (5.4%) IgG, ANDV	Healthy general population	[58]
Brazil (Amazon)	All samples were negative for IgM and 10/1731 (0.6%) for IgG, ARAV	Healthy general population	[59]

^a^ Immunoglobulin testes was IgG, except otherwise specified; ^b^ All the studies used SNV antigen except otherwise specified.
